# Long-term study of the prevalence of *Borrelia burgdorferi* s.l. infection in ticks (*Ixodes ricinus*) feeding on blackbirds (*Turdus merula*) in NE Poland

**DOI:** 10.1007/s10493-016-0082-x

**Published:** 2016-09-08

**Authors:** Alicja Gryczyńska, Renata Welc-Falęciak

**Affiliations:** 1 Department of Ecology, Institute of Zoology, Faculty of Biology, Biological and Chemical Research Centre, University of Warsaw, 101 Żwirki i Wigury St., 02-089 Warsaw, Poland; 2 Department of Parasitology, Institute of Zoology, Faculty of Biology, University of Warsaw, 1 Miecznikowa St., 02-096 Warsaw, Poland

**Keywords:** Blackbirds, *Borrelia burgdorferi* s.l., *Ixodes ricinus*, Lyme borreliosis

## Abstract

Seeking evidence to confirm that blackbirds (*Turdus merula*) may be involved in environmental maintenance of *Borrelia burgdorferi* s.l. (the causative agent of Lyme borreliosis), we conducted a long-term study over three separate 2-year periods, together embracing a span of almost 20 years, all in the same area in northeastern Poland. We examined a total of 78 blackbirds and collected 623 *Ixodes ricinus* ticks feeding on them. The tick infestation prevalence was found to be very high (89.7 %). Among all ticks collected, 9.8 % individuals were infected with *B. burgdorferi* s.l. spirochetes. We found statistically significant growth in the prevalence of infected ticks as well as an increasing proportion of blackbirds hosting them in subsequent years of study. Ticks feeding on blackbirds were infected mainly with *B. garinii* (45.7 %), a genospecies commonly encountered in birds, and with *B. afzelii* (28.6 %), until recently considered rodent-associated. We also identified *B. turdi* (22.9 %), frequently found in recent years in ticks feeding on birds, and *B. spielmanii* (2.8 %), which had previously not been found in infected ticks feeding on blackbirds. We also found that ticks infected with genospecies associated with avian reservoir groups (*B. garinii* and *B. turdi*) were not randomly distributed on blackbirds, but instead focused on certain bird specimens. We therefore conjecture that this is a result of ticks becoming infected either from the host blackbird itself, or from other infected ticks feeding on the same host blackbird. We did not find any similar dependency for the rodent specialist *B. afzelii*.

**Electronic supplementary material:**

The online version of this article (doi:10.1007/s10493-016-0082-x) contains supplementary material, which is available to authorized users.

## Introduction

*Borrelia burgdorferi* sensu lato (s.l.) is a genospecies complex of Gram-negative spirochete bacteria with a worldwide distribution. Of the 20 genospecies that have been identified to date, nine (*B. afzelii, B. bavariensis, B. bissettii, B. burgdorferi* s.s., *B. garinii, B. kurtenbachii, B. lusitaniae, B. spielmanii* and *B. valaisiana*) infect humans, causing Lyme borreliosis, which is a multisystem, tick-borne zoonosis with a wide spectrum of clinical manifestations (Margos et al. 2011; Rudenko et al. 2011; Ivanova et al. 2014). The bacterium is transmitted by ticks from the Ixodidae family (in Europe mainly *Ixodes ricinus*) and is maintained in nature by at least 237 animal species, which are available to serve as its potential reservoir hosts (Gern 2008). The ixodid ticks have low mobility and are mainly dispersed by their host species. In their life cycle, ticks require vertebrate hosts, most frequently mammals, of which rodents and insectivores are the core group of hosts for immature ticks (larvae and nymphs), whereas carnivores and ungulates are the main hosts chosen by adult ticks (Kjelland et al. 2011; Radzijevskaja et al. 2013). However, while carrying a heavy tick burden, mammals can only disperse feeding ticks over short- and medium-range distances. Birds are the second group of vertebrates on which ixodid ticks can engorge. They, especially migratory species, may transport ticks over long distances and across geographical barriers, such as mountains, deserts or oceans, which would halt mammals. Many bird species throughout the world are infested with ticks and act as transport vehicles for these ectoparasites across a continent or from one continent to another during their seasonal migration (Olsen et al. 1995b; Smith et al. 1996; Gryczyńska et al. 2002; Hasle et al. 2009).

Hence, birds are potential disseminators of *B. burgdorferi* s.l. spirochete, either as carriers of infected ticks or as reservoir hosts of the pathogen. Birds contribute to the transmission of *B. burgdorferi* s.l. in nature via two different enzootic cycles: one marine, the other terrestrial (Humair 2002). The former cycle occurs in marine environments and involves seabirds (e.g. albatrosses, guillemots, petrels, razorbills, gulls, puffins, kittiwakes—Olsen et al. 1995a; Gylfe et al. 1999; Gasparini et al. 2001). The latter cycle involves ground-dwelling birds such as game birds (e.g. quails, pheasants—Isogai et al. 1994a; Kurtenbach et al. 1998a, b) and passerines (Gryczyńska et al. 2004; Dubska et al. 2009, 2011; Marsot et al. 2012; Geller et al. 2013; Hasle 2013; Lommano et al. 2014). Some studies have focused on this *B. burgdorferi* s.l. dissemination potential of different passerine bird species within a community during their breeding season (Gryczyńska et al. 2002; Marsot et al. 2012; Lommano et al. 2014). In this case, birds are sedentary and settled in their reproductive territories, but the relative contribution of each passerine bird species in tick hosting and survival of *B. burgdorferi* s.l. differs and depends on many factors. Differences in behavior among passerine bird species is regarded as the main cause of this inter-specific variation (Marsot et al. 2012). Ground-dwelling passerine bird species were found to carry a higher tick burden than bird species that do not forage and nest close to the ground (ticks are generally found on vegetation within 2 m of the ground). Numerous studies have shown that members of the thrush family (Turdidae), such as the blackbird (*Turdus merula*) and the song thrush (*Turdus philomelos*), which tend to be ground-dwelling species, were more heavily infested with ticks than other bird species (Gryczyńska et al. 2002; Michalik et al. 2008; Marsot et al. 2012; Norte et al. 2015).

This kind of ground-dwelling behavior of the blackbird was what prompted us to study this species and its role in the ecology of Lyme borreliosis in Poland. Blackbirds are common in forests, widespread in bushes and green areas of rural environments, and have recently colonized urban areas with great success. The blackbird’s particularly intensive involvement as a tick host, and therefore also its role as a competent reservoir of vector-borne zoonotic pathogens, has caused it to become a very important species in terms of epidemiology and veterinary science. In our long-term study, which was conducted over three different 2-year periods, together spanning almost 20 years, in the same area in northeastern Poland considered to be particularly densely populated by ticks and various pathogens transmitted by them, we sought evidence to support the hypothesis that birds, especially the true thrushes (Turdus spp.), are growing in importance as *B. burgdorferi* s.l. disseminators.

## Materials and methods

### Study site, tick collection

The Mazurian Lake region, located in northeastern Poland (53°47′N, 21°34′E), was selected as the study site. Investigations were performed in a wooded area. Birds were captured using mist nets. Only blackbirds, belonging to the family Turdidae, were included in this study. The catches were performed during three non-consecutive, 2-year periods (1996–1997; 2005–2006; 2011–2012), in the season of highest tick activity in June. Captured blackbirds were ringed, their age and sex were determined as per Svensson (1992), and they were visually examined for the presence of ticks. The body of each individual was scanned by moving or blowing the feathers to see the skin. Ticks were found only on the birds’ heads (around the eyes, ears and beak). All ticks found were removed with forceps and placed in tubes containing 70 % ethanol; subsequently their species and life stages were identified in the laboratory (Siuda 1993). The blackbirds were released into the same habitats in which they were caught. All the procedures were approved by the First Warsaw Local Ethics Committee for Animal Experimentation (permission no. OP 4072/230/97, 640/2006).

### Laboratory procedures

Genomic DNA was extracted from whole ticks. In the first two investigation periods (1996–1997 and 2005–2006), the DNA was isolated following the procedure of Rijpkema et al. (1996), whereas in the third period (2011–2012), in line with advances in common practice in the field, genomic DNA was extracted from homogenized tick specimens using the DNA easy Blood and Tissue Kit (Qiagen, Crawley, UK), in accordance with the manufacturer’s instructions. To check whether amplifiable DNA had been extracted, for both isolation methodologies we used PCR reactions employing tick-specific 18S rRNA primers (Mangold et al. 1998; Noureddine et al. 2011). Only positive samples were chosen for further analysis. All the samples were stored at −20 °C.

DNA amplification was performed using the nested PCR technique according to Valsangiacomo et al. (1996), based on two-stage amplification of the fragment of the *hbb* gene coding a highly preserved histon protein of *B. burgdorferi* s.l. The first-stage product was 433 base pairs in size and contained the whole *hbb* gene, and the second-stage product was a 184 base-pair fragment of the *hbb* gene. The cycling conditions and PCR primer sequences were published by Valsangiacomo et al. (1996): denaturation at 94 °C for 60 s, annealing at 52 °C for 60 s and then extending at 72 °C for 30 s for a total of 35 cycles in the first stage and 25 cycles in the second stage (I PCR *hbb* for: GCGAAGAATTCATAAAAATAAGGCTGC, *hbb* rev: TATAAGAATTCACGATATTAACTGGC; II PCR *hbb* for: AGATGCTTTTTTTGAAGAGC, *hbb* rev: CAAATCTTTGCCTGGACG). Genomic DNA of *B. burgdorferi* s.l. (DNA-Gdańsk II, Poland and Imogena) was used as a positive control, and reagents as a negative control. The second-stage products were separated by electrophoresis in 2 % agarose gel in TAE buffer and visualized by ethidium bromide at 0.5 µg/ml staining. DNA was analyzed in UV light, wavelength 300 nm (BioRad).

Ticks collected in 2011–2012 were further analyzed with additional PCR reaction. Detection and genotyping of *B. burgdorferi* s.l. were performed by amplification and sequencing of the 357 base-pair fragment of 16S rRNA gene. The thermal profiles and PCR primer sequences used in this study had been previously described by Marconi and Garon (1992): denaturation at 94 °C for 60 s, annealing at 48 °C for 30 s and extending at 72 °C for 90 s for a total of 40 cycles (LD for: GACTTATCACCGGCAGTCTTA, LD rev: ATGCACACTTGGTGTTAACTA). Amplicons were visualized with Midori Green stain (Nippon Genetics Europe GmbH) following electrophoresis in 2 % agarose gels. Amplicons were purified using the Axygen Clean-up purification kit (Axygen, USA) and sequenced by a private company (Genomed S.A., Poland) in both directions. In order to compare nucleotide sequences with data stored in GenBank databases (http://www.ncbi.nih.gov/Genbank/index.html), BLAST-NCBI programs were used (http://www.ncbi.nlm.nih.gov/BLAST/). The nucleotide identity/similarity of the sequenced 16S rDNA fragments of each *Borrelia* species was very high (99.5–100 %). The nucleotide sequences of isolates from *B. garinii* were identical to the sequences obtained from birds (*Fratercula arctica* [AJ009749]) and ticks (*I. ricinus* [GQ918150, AM418453] *I. persulctus* [EF488991], *I. uriae* [AJ009749]). The nucleotide sequences of *B. afzelii* isolates were closely related (>99 %) to *B. afzelii* obtained from *I. perculcatus* in Russia [CP009212] and from humans in China [NR074662]. *Borrelia turdi* isolates showed 99.3 % similarity to *B. turdi* originally isolated from Ixodid ticks in Japan [AM418453]. The sequence of the *Borrelia spielmanii* group was closely related (98.9 %) to the *B. spielmanii* strain DSM 16813T [HE582779].

### Statistical analysis

We performed a Chi square test to compare the percentages. The mean values and correlations were compared using the Generalized Linear Model (GLM) procedure. Calculations were carried out using the scripting language R. The significance of the clustering of infected ticks on the same bird was evaluated using a simulation test written in R script (given in ESM Appendix). We performed 100,000 iterations of the simulation.

## Results

A total of 623 ticks engorged on 78 blackbirds were examined during the 17-year period. All of them belonged to the *Ixodes ricinus* species. The tick infestation prevalence was found to be 89.7 % and ranged from 78.6 % in 1997 to 100 % in 2006 (Table 1). There were no significant differences in tick prevalence between years of study (*χ* ^2^ = 4.955; *P* = 0.12). The tick infestation intensity averaged eight specimens per bird, ranging from 4.2 in 1997 to 14.2 in 2005 (Table 1). The highest number of *I. ricinus* specimens found feeding on a single blackbird was 39. A statistically significant dependence was found between the mean number of ticks feeding on a bird and the year of study (*F* = 2.83; *P* < 0.03). All the blackbird-engorged ticks were in a young, predominantly nymph stage, with 131 larvae (21 %) and 492 nymphs (79 %) identified (Table 1). Neither the prevalence (*χ* ^2^ = 1.326; *P* = 0.56) nor intensity of tick infestation (*F* = 1.19; *P* = 0.31) showed any correlation with the age or sex of the bird.

**Table 1:** *Borrelia burgdorferi* s.l. infection in ticks infesting blackbirds resident in NE Poland

Year	No. of birds examined	No. of birds infested by ticks/prevalence (%)	No. of ticks collected (larvae; nymphs)/intensity (ticks per bird) [±SD]	No. of ticks with *B. burgdorferi* s.l. (larvae; nymphs)/prevalence (%)	No. of birds infested by ticks with *B. burgdorferi* s.l./prevalence (%)
1996	11	10/90.9	58 (23; 35)/5.3 [±6.2]	5 (0; 5)/8.6	3/27.3
1997	14	11/78.6	59 (9; 50)/4.2 [±5.6]	4 (0; 4)/6.8	3/21.4
2005	14	13/92.8	199 (71; 128)/14.2 [±13.6]	8 (1; 7)/4	4/28.6
2006	15	15/100	158 (15; 143)/10.5 [±7.1]	9 (1; 8)/5.7	5/33.3
2011	14	13/92.8	103 (10; 93)/7.4 [±9.9]	21 (0; 21)/20.4	5/35.7
2012	10	8/80	46 (3; 43)/4.6 [±4.3]	14 (2; 12)/30.4	4/40
Total	78	70/89.7	623 (131; 492)/8 [±9.1]	61 (4; 57)/9.8 (3; 11.6)	24/30.8

Among all ticks collected from blackbirds, 61 (9.8 %) individuals were infected with *Borrelia burgdorferi* s.l. bacterium. The lowest percentage of ticks infected was found in 2005, the highest in 2012: 4 and 30.4 %, respectively (Table 1). Most of the infected ticks were at the nymph stage; the infection rate among larvae and nymphs was 3 and 11.6 %, respectively (*χ* ^2^ = 14.31; *P* < 0.001) (Table 1). Among the group of blackbirds particularly heavily infested—with more than ten ticks per bird—we found a statistically significantly higher percentage of ticks infected with *Borrelia burgdorferi* s.l. (*n* = 443; 11.5 %) than among the group of birds with smaller numbers of ticks feeding on them (*n* = 180; 5.56 %) (*χ* ^2^ = 5.14; *P* < 0.02).

Statistically significant differences were found between particular years of the study in terms of the infection of ticks feeding on blackbirds (*χ* ^2^ = 46.49; *P* < 0.001). Additionally, statistically significant growth in the prevalence of infected ticks was demonstrated in given years of the study (*r* = 0.696; *F*_4,1_ = 3.759; *P* < 0.05). The percentage of blackbirds hosting ticks infected by *B. burgdorferi* s.l. was also found to increase significantly in subsequent years of the study (*r* = 0.91; *F*_4,1_ = 18.51; *P* < 0.005) (Table 1). Such a tendency was observed for blackbirds hosting both infected larvae and infected nymphs, and this growth was evaluated as statistically significant (GLM; *F* = 7.66; *P* < 0.025).

**Fig. 1:**
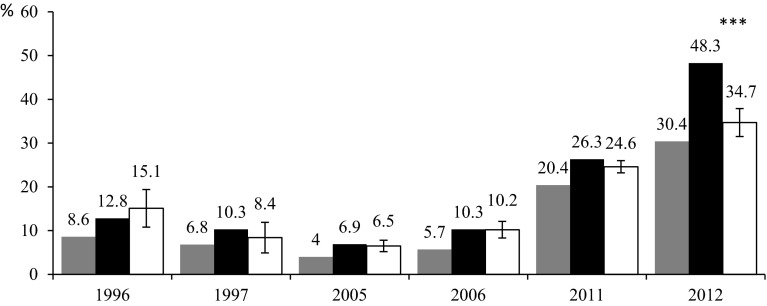
A comparison of *Borrelia burgdorferi* s.l. prevalence in all ticks feeding on blackbirds, in particular years, with the prevalence of *B. burgdorferi* s.l. in ticks feeding on blackbirds hosting at least one infected tick. *Grey bars B. burgdorferi* s.l. prevalence in all ticks feeding on blackbirds; *Black bars B. burgdorferi* s.l. prevalence in ticks feeding on blackbirds hosting at least one infected tick; *White bars* mean (±SD) *B. burgdorferi* s.l. prevalence in ticks feeding on blackbirds hosting at least one infected tick at randomly distributed infected ticks on birds; *asterisks* indicate significant difference between black and white bars

The percentage of infected ticks feeding on blackbirds was calculated both for all blackbirds trapped and for only those blackbirds that hosted at least one infected tick (Fig. 1). Additionally, the mean percentages of infected ticks for randomly distributed infected ticks among birds were calculated to check whether the infected ticks were focused on certain birds. Comparison of these two figures in particular years of study showed that in 2012 *B. burgdorferi* s.l. positive ticks were not randomly distributed on birds (Fig. 1, simulating test, *P* < 0.002, see ESM Appendix).

**Fig. 2:**
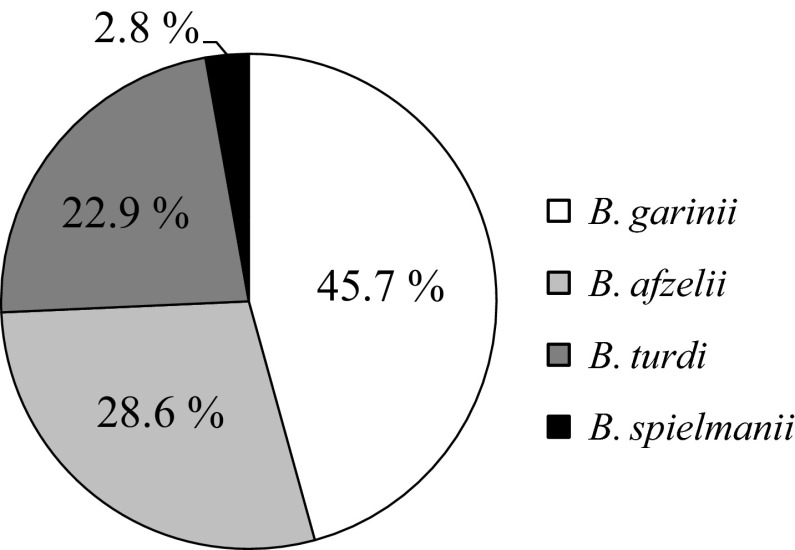
The prevalence of *Borrelia burgdorferi* respective genospecies stated in ticks feeding on blackbirds

Ticks feeding on blackbirds collected in 2011 and 2012 (*n* = 35) were infected with 4 *B. burgdorferi* s.l. genospecies—*B. garinii* (*n* = 16; 45.7 %), *B. afzelii* (*n* = 10; 28.6 %), *B. turdi* (*n* = 8; 22.9 %), and *B. spielmanii* (*n* = 1; 2.8 %). The prevalence of the first three was similar in both study years, but *B. spielmanii* infected only one tick specimen in 2011 (Fig. 2). Among the infected ticks, only two were in the larvae stage, and they were found to be carrying exclusively the *B. garinii* genospecies. In the case of blackbirds with more than one infected tick, two, three or four different genospecies of *B. burgdorferi* were found to be present in those ticks. However, no individual tick was found to be infected with more than one genospecies of the bacterium.

**Fig. 3:**
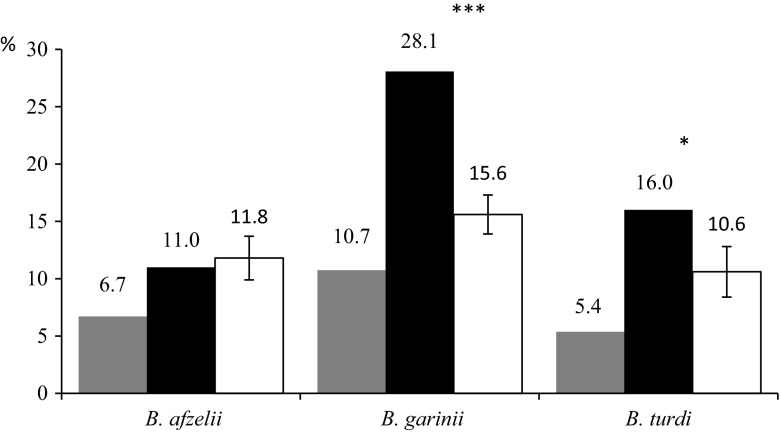
A comparison of *Borrelia burgdorferi* respective genospecies prevalence in all ticks feeding on blackbirds with the prevalence of them in ticks feeding on blackbirds hosting at least one infected tick. *Grey bars B. burgdorferi* respective genospecies prevalence in all ticks feeding on blackbirds; *Black bars B. burgdorferi* respective genospecies prevalence in ticks feeding on blackbirds hosting at least one infected tick; *White bars* mean (±SD) *B. burgdorferi* respective genospecies prevalence in ticks feeding on blackbirds hosting at least one infected tick at randomly distributed infected ticks on birds; *asterisks* indicate significant difference between black and white bars

The proportion of ticks infected with one of three different genospecies (*B. garinii*, *B. afzelii*, *B. turdi*) feeding on blackbirds was calculated both for all blackbirds trapped and for only those blackbirds that hosted at least one infected tick (Fig. 3). Additionally, the mean proportions of infected ticks for randomly distributed infected ticks among birds were calculated to check whether the infected ticks focused on certain birds. Comparison of these two figures showed that *B. garinii* and *B. turdi* positive ticks were not randomly distributed on birds (simulating test, respectively *P* < 0.001 and *P* = 0.03, see ESM Appendix). For *B. afzelii* no such dependency was found (simulating test, *P* = 0.6) (Fig. 3).

## Discussion

Long-term studies evidencing trends in the relations between ticks, tick-borne pathogens and their vertebrate hosts are very important for understanding the etiology of many human diseases. Certain potential changes in a vertebrate’s role in maintenance of causative agents of illnesses may become observable only in longitudinal surveys carried out over many years. However, in the existing literature there is only one study, by Hasle et al. (2009), comparing data on prevalence of tick infestation on migrating passerine birds. These authors compared data collected from 2003 to 2005 with data previously published by Mehl et al. (1984), collected between 1965 and 1970 by two Norwegian bird observatories, and found a significant increase in prevalence of ticks in migratory passerine birds. In our study, we demonstrated that the prevalence of tick infestation in blackbirds was very high. In all years of investigation, at least 78.6 % individuals were tick carriers; in certain years, ticks were found on all the blackbirds caught. With such great prevalence, it is not possible to determine any statistically significant differences depending on a bird’s age or sex. On the other hand, the intensity of blackbird infestation was shown to differ significantly in consecutive years of study, which was probably related to tick densities in the habitat studied in the respective years of study.

Some behaviors on the part of blackbirds and some aspects of their biology make them more vulnerable to contact with ticks than other passerine birds. The mean foraging (2.5 m) and nesting (1.6 m) heights for the species are in the tick dwelling zone, meaning that blackbirds are in continuous interaction with ticks (Marsot et al. 2012). The presence of feeding ticks and particularly high intensity of infestation have some impact on the health indicators of their avian hosts, possibly causing anemia, mass loss and low body condition. Norte et al. (2013c) found that infestation by ticks significantly increased certain physiological variables in blackbirds, and also caused a significant worsening of body condition in song thrushes, suggesting increased stress levels in the birds investigated. Studying passerine bird communities, Marsot et al. (2012) found that all thrush species investigated (blackbird, song thrush and mistle thrush) have the greatest tick burdens and also relatively large spleens for a given body size. This result corresponds with Møller and Erritzøe’s (2002) findings that a relatively large spleen is associated with high impact of ectoparasites in birds. We therefore conjecture that the individual blackbirds we studied may vary in terms of their susceptibility to infection with *B. burgdorferi* s.l. bacteria. Those individuals which have many ticks feeding on them not only stand greater chances of contact with pathogens carried by the latter, but may, as a result of the strong invasion of ectoparasites, also be more susceptible to infection and more likely to pass on pathogens to other ticks feeding on them. In our study, we found a significantly higher percentage of infected ticks among those feeding on blackbirds with the highest level of infestation, as compared to ticks from birds not attacked by many parasites. This was found to be true despite the fact that the lowest level of *B. burgdorferi* s.l. infection prevalence was ascertained in the years when blackbirds were infested most intensively (2005, 2006).

We found that *B. burgdorferi* s.l. infection in ticks engorged on blackbirds has increased very significantly in recent years, in comparison with previous study periods. Presumably, this is closely connected to a steady rise of infection both in ticks seeking a host and ones feeding on small rodent reservoir hosts in the environment (Siński et al. 2006). Another possible reason is the growing overall percentage of passerine birds, mainly blackbirds or other thrushes, infected with pathogens at our study site. This hypothesis is supported by the fact that we saw a continuous increase in the proportion of blackbirds carrying *B. burgdorferi* s.l. infected ticks in consecutive years of the study. As is presently known, birds can serve as *B. burgdorferi* s.l. competent reservoirs in the environment, and ticks become infected as a result of engorging on infected birds (Norte et al. 2013a, b). Birds, as opposed to small rodents, can live even a number of years in natural circumstances. Even though small rodents constitute the main competent reservoir of *B. burgdorferi* s.l. in the environment, their populations see a significant decline in numbers in winter, and an intensive advent of young, pathogen-free individuals in early spring. Thus, the role of birds—both sedentary species, always present in particular area, and migratory ones, most often coming back to their reproduction habitat every year—as the main pathogen source for consecutive tick generations significantly increases in the post-winter period in a given area. Not only do winter and spring constitute periods when there is a significant shortage of small mammals, which are the main group of hosts for immature ticks, as well as a competent reservoir for *B. burgdorferi* s.l. bacteria; the concentration of such mammals in a given area may also vary greatly across successive years. The population dynamics of small rodents from year to year is closely related to factor including food availability (Pucek et al. 1993). Jones et al. (1998) presented an example of a network of dependencies comprised of many factors affecting the risk of *B. burgdorferi* s.l. infection in black-legged ticks (*Ixodes scapularis*) in subsequent years. One factor contributing to the increased risk of infection is the density of population of white-footed mouse (*Peromyscus leucopus*) and white-tailed deer (*Odocoileus virginianus*), constituting a group of hosts for ticks and a competent reservoir for *B. burgdorferi* s.l. bacteria. This parameter in turn is closely dependent upon the availability of acorns produced by oak trees and the potentially heavy impact of the gypsy moth (*Lymantria dispar*) on them.

In our study site, we also saw long-term dynamics in small mammal communities, as populations responded to food availability (Kozakiewicz and Kozakiewicz 2008). These authors found a nearly tenfold difference in the density of individual rodent species in different years of study. Unlike small mammals, the populations of passerine birds in our study site did not show such large fluctuations in successive years. Based on long-term observations carried out in the area, we know that blackbird numbers are relatively stable. This is in large part related to the type of food preferred by the species (mainly invertebrates and fruit) and the strong territorial behavior that the birds exhibit during the breeding season (a major share of the time the species spends in the study area). We can assume that this means that in years when lower densities of small rodents are observed, blackbirds may account for a greater proportional share as hosts for immature tick stages and as a competent reservoir for *B. burgdorferi* s.l. bacteria as compared to years when there are many rodents, when the latter constitute the main group of animals attacked by such ticks. Apart from small mammals and passerine birds, our study site is also home to sand lizards (*Lacerta agilis*), which likewise represent a host group for tick larvae and nymphs. However, the tick infestation rate in their case, at 13.2–40.4 % (Gryczyńska-Siemiątkowska et al. 2007; Ekner et al. 2011), is relatively low as compared to that of mammals and passerine birds.

There are several different schemes of immune system functioning and *B. burgdorferi* s.l. survival ability in the respective vertebrate groups. Due to differences in their serum complement sensitivity, some *B. burgdorferi* s.l. genospecies are known to be associated with different reservoir groups (Kurtenbach et al. 1998b). It has been clearly demonstrated that birds are competent reservoir hosts for *B. garinii*, *B. valaisiana* and *B. burgdorferi* s.s. (Derdáková and Lenčáková 2005; Michalik et al. 2008; Kempf et al. 2011; Norte et al. 2013b). In the infected ticks collected from birds in our study in 2011 and 2012, a marked prevalence of the *B. garinii* genospecies was found. The remaining genospecies associated with this group of vertebrates, i.e., *B. valaisiana* and *B. burgdorferi* s.s., were not discovered. Kipp et al. (2006), in their study in Germany, also found *B. garinii* to be the most frequent genospecies in ticks feeding on blackbirds (87.5 %), with no ticks found on that bird species carrying *B. valaisiana* and *B. burgdorferi* s.s. In investigations carried out in west-central Poland, Michalik et al. (2008) showed that, in infected ticks feeding on blackbirds, *B*. *garinii* was the most frequent, with a small addition of *B. valaisiana* (95 and 5 %, respectively). A much higher proportion (75 %) of *B. valaisiana* in ticks engorging on blackbirds was determined by Hanincová et al. (2003) in Slovakia. The remaining 25 % of ticks in that study were infected by *B. garinii.* Similarly, in a study conducted in Latvia, among infected ticks feeding on blackbirds, 9.1 % were infected only with *B. garinii*, the majority (54.5 %) showing *B. valaisiana* infection and the remainder (36.4 %) being coinfected with both these genospecies (Capligina et al. 2014).

The genospecies *B. afzelii* was, until recently, considered a rodent specialist occurring only in ticks engorging on mammals, mainly on small rodents, and not infective for birds (Hanincová et al. 2002). Studies found a high percentage of *B. afzelii* infection in ticks seeking a host in a habitat, whereas this genospecies was absent in infected ticks feeding on birds living there (Hanincová et al. 2003; Michalik et al. 2008). It was considered a rodent-associated genospecies, eliminated from ticks feeding on avian hosts as a result of their sensitivity to the host complement (Kurtenbach et al. 2002). This opinion, however, has recently been altered by findings that birds can effectively maintain *B. afzelii* genospecies (Dubska et al. 2009; Franke et al. 2010; Geller et al. 2013). Dubska et al. (2009) state that, in infected ticks feeding on blackbirds, *B. afzelii* genospecies constituted 1.9 %, whereas Taragelová et al. (2008) reported the same percentage for blackbirds to be 4.8 %. In the investigation conducted by Kipp et al. (2006) in Germany, blackbirds were also infested by ticks infected with *B. afzelii,* but its proportion among other genospecies amounted to 12.5 %. The important role of blackbirds in transmitting *B. afzelii* is also supported by the fact that Franke et al. (2010) found tick larvae infected with this genospecies. In our work, we discovered larvae infected only with *B. garinii.* However, the total percentage of *B. afzelii* in all feeding ticks, as shown in our study, amounted to 28.6 %, which would confirm the hypothesis that blackbirds are becoming carriers of ticks infected with this rodent specialist as well, and in future may constitute a competent reservoir for it at the study site.

The *B. lusitaniae* genospecies, on the other hand, considered to be associated with lizards (Richter and Matuschka 2006; Ekner et al. 2011; Norte et al. 2014), was not detected in the bird-engorged ticks examined in our study.

Birds have recently been demonstrated to play an important role as reservoirs and dispersers of *B. turdi* (Norte et al. 2013a, b). In our study, a comparatively high percentage of ticks were found to be infected by this genospecies. *B. turdi* used to be considered uncommon in Europe, but it has been frequently found in ticks feeding on birds in recent years, mainly in Portugal (Norte et al. 2013a, b, 2014). Norte et al. (2015) showed only *B. turdi* infection in *I. ricinus* ticks feeding on blackbirds. A xenodiagnostic experiment confirmed the role of blackbirds as competent reservoirs for *B. turdi* and *B. valaisiana* (Norte et al. 2013a).

The *B. spielmanii* genospecies has so far not been reported in infected ticks feeding on blackbirds. Moreover, the result obtained in our work represents the first evidence for the presence of *B. spielmanii* not only in ticks feeding on blackbirds, but in ticks feeding on birds in general.

In our study, we also found that *B. burgdorferi* s.l. positive ticks were not randomly distributed on birds. This was seen in 2012, when infected ticks were largely focused on certain blackbirds. We therefore further evaluated also the distribution of ticks infected with individual genospecies of bacteria and found that ticks infected with *B. garinii* and *B. turdi* were not randomly distributed on birds. Instead, such ticks were found to be clustered on selected individual blackbirds. We can therefore conjecture that this is the result of ticks becoming infected from the blackbird itself, or from other ticks feeding on it. This finding may suggest that, in the study site environment, the blackbirds act as a competent reservoir of *B. burgdorferi* s.l. genospecies associated with avian reservoir groups and allow pathogen transmission to ticks engorged on them. We did not find any such dependency for ticks infected with *B. afzelii.* Even though blackbirds in the study site are carriers of ticks infected with this rodent specialist, they still cannot be recognized as also being involved in the spread of this genospecies.

Our study site is inhabited by several larger-sized vertebrate species that constitute a group of hosts not only for immature but also for adult ticks. These are mainly representatives of Carnivora, such as red foxes (*Vulpes vulpes*), raccoon dogs (*Nyctereutes procyonoides*), European badgers (*Meles meles*), European pine martens (*Martes martes*) and European minks (*Mustela lutreola*). However, it has been shown that only raccoon dogs can serve as a competent reservoir for the bird specialist *B. garinii.* In a study conducted in western Poland, the rate of raccoon dog infection with this genospecies was found to be 62.5 % (Wodecka et al. 2016). In contrast, *B. afzelii*, *B. valaisiana* and *B. burgdorferi* s.s. infection was ascertained in all investigated carnivores and ticks engorged on them (Isogai et al. 1994b; Gern and Sell 2009; Gherman et al. 2012; Wodecka et al. 2016). We can conjecture, therefore, that the appearance of a raccoon dog population in Poland 60 years ago and its successful spread in forest habitats (Nowak and Pielowski 1964) may have contributed to the maintenance of *B. garinii* in the environment, and therefore also to the incorporation of blackbirds in the transmission cycles for this genospecies and the increasing role of this bird species as a competent reservoir for it in the study site over the years.

This strongly justifies the continuance of our long-term study into this problem, and furthermore suggests that we should also begin to investigate *B. burgdorferi* s.l. infection in blackbirds’ blood, then collate this data with historical data available for the same area. Such a study was conducted almost 20 years ago, and the prevalence of infection in blackbirds’ blood was ascertained as 4.2 % (Gryczyńska et al. 2004); since then no study involving birds’ blood infection has been carried out in the given area. Only Michalik et al. (2008) showed 2 % *B. burgdorferi* s.s. infection in blood of song thrush captured in west-central Poland.

The majority of *B. burgdorferi* s.l. genospecies which the blackbird-engorged ticks were found to be carrying are known to be pathogenic for humans, causing Lyme borreliosis (Rudenko et al. 2011). This makes studies on this group of vertebrates extremely important in terms of epidemiology and veterinary science. Wild birds increasingly colonize parks, gardens and green spaces within cities, with a growing number of migrating passerine species finding suitable conditions for living there, and even for spending winters. The blackbird is a good example of such a species, as it is known to be undergoing an intensive urbanization process in many European cities, with its population being divided into rural (migrating) and urban (sedentary) (Evans et al. 2010). Taking into account its common occurrence, behavior, and even certain features of its anatomy and physiology, it is presently considered one of the bird species most highly involved in the survival of *B. burgdorferi* s.l. bacterium in the environment.

In conclusion, our study results highlight the increasing role of blackbirds as carriers of ticks infected with *Borrelia burgdorferi* s.l. genospecies of epidemiologic importance in the natural environment. Relative growth in the relevance of birds in relation to other groups of vertebrates constituting competent reservoirs for this pathogen is detectable only in long-term studies, thus longitudinal surveys such as this one, carried out over a number of years, seem to be a more reliable method for predicting further dynamics in this regard than cross-sectional analyses. Also, the increasing role of blackbirds as a source and potential disseminator of vector-borne zoonotic pathogens ought to be considered in future investigations, taking into account their colonization of urban areas.

## Electronic supplementary material

Below is the link to the electronic supplementary material.

Supplementary material 1 (TXT 0 kb)
